# Retraction

**DOI:** 10.1111/cas.15305

**Published:** 2022-04-08

Liming Li, Qingfeng Zhang, Xin Wang, Yan Li, Huifen Xie, Xiangdong Chen, Squalene epoxidase‐induced cholesteryl ester accumulation promotes nasopharyngeal carcinoma development by activating PI3K/AKT signaling, Cancer Science 2020, 111 (7), pp. 2275–2283 (https://onlinelibrary.wiley.com/doi/full/10.1111/cas.14426).

The above article, published online on 21 April 2020 in Wiley Online Library (wileyonlinelibrary.com), has been retracted by agreement between the authors, Liming Li, Qingfeng Zhang, Xin Wang, Yan Li, Huifen Xie, Xiangdong Chen, the journal Editor in Chief, Masanori Hatakeyama, Japanese Cancer Association, and John Wiley and Sons Australia, Ltd. The authors reported that Figure 6F and 6B do not reflect the results of the experiments described. Further, following additional experiments, the authors were unable to reproduce the results shown in Figure 6C. These issues affect the conclusions of the study and a retraction has been agreed.

